# Role of Nse1 Subunit of SMC5/6 Complex as a Ubiquitin Ligase

**DOI:** 10.3390/cells11010165

**Published:** 2022-01-04

**Authors:** Peter Kolesar, Karel Stejskal, David Potesil, Johanne M. Murray, Jan J. Palecek

**Affiliations:** 1 National Centre for Biomolecular Research, Faculty of Science, Masaryk University, 62500 Brno, Czech Republic; 2 Central European Institute of Technology, Masaryk University, 62500 Brno, Czech Republic; karel.stejskal@imba.oeaw.ac.at (K.S.); david.potesil@ceitec.muni.cz (D.P.); 3 Genome Damage and Stability Centre, School of Life Sciences, University of Sussex, Falmer, Brighton BN1 9RH, UK; j.m.murray@sussex.ac.uk

**Keywords:** SMC5/6, Nse1, ubiquitin ligase, ubiquitination, Ubc13/Mms2, Nse4 kleisin

## Abstract

Structural Maintenance of Chromosomes (SMC) complexes are important for many aspects of the chromosomal organization. Unlike cohesin and condensin, the SMC5/6 complex contains a variant RING domain carried by its Nse1 subunit. RING domains are characteristic for ubiquitin ligases, and human NSE1 has been shown to possess ubiquitin-ligase activity in vitro. However, other studies were unable to show such activity. Here, we confirm Nse1 ubiquitin-ligase activity using purified *Schizosaccharomyces pombe* proteins. We demonstrate that the Nse1 ligase activity is stimulated by Nse3 and Nse4. We show that Nse1 specifically utilizes Ubc13/Mms2 E2 enzyme and interacts directly with ubiquitin. We identify the Nse1 mutation (R188E) that specifically disrupts its E3 activity and demonstrate that the Nse1-dependent ubiquitination is particularly important under replication stress. Moreover, we determine Nse4 (lysine K181) as the first known SMC5/6-associated Nse1 substrate. Interestingly, abolition of Nse4 modification at K181 leads to suppression of DNA-damage sensitivity of other SMC5/6 mutants. Altogether, this study brings new evidence for Nse1 ubiquitin ligase activity, significantly advancing our understanding of this enigmatic SMC5/6 function.

## 1. Introduction

Structural Maintenance of Chromosomes (SMC) complexes cohesin, condensin, and SMC5/6, are important for many aspects of the chromosomal organization, dynamics, and stability of the genome [1]. These complexes are scaffolded by dimers of SMC ATPases with long arms, which can encircle DNA. In eukaryotes, the Smc1-Smc3 heterodimer forms the core of the cohesin complex that ensures proper segregation and recombination of sister chromatids. The Smc2-Smc4 heterodimer forms the core of the condensin complex that promotes chromosome condensation during mitosis. The SMC5/6 complex has less well-defined roles, but it is also essential. It was first identified based on its role in dealing with ionizing and ultraviolet radiation [2]. Since then, SMC5/6 has been best known for its role in DNA repair by homologous recombination (HR), where it is thought to have both an early and a late function [3]. SMC5/6 is also important for timely replication of the ribosomal DNA array, stabilization of stalled replication forks, maintenance of the checkpoint and heterochromatin, accurate chromosome segregation, and inhibition of transcription (reviewed in [4,5]).

The Smc5-Smc6 heterodimer forms the SMC5/6 complex along with six Nse (non-Smc element) subunits [6]. The Nse4 kleisin molecule binds SMC6 neck and SMC5 cap via its N- and C-terminal domains, respectively, analogously to the other SMC complexes [7,8]. Similar to prokaryotic SMC complexes, the Nse1-Nse3 kite subunits bind the Nse4 middle region via their winged-helix B (WHB) domains and regulate the dynamics of the SMC5/6 ATPase cycle [8,9,10,11,12,13]. Uniquely for SMC complexes, Nse1 and Nse2 subunits possess enzymatic activities (reviewed in [4,14]). Nse2 contains an SP-RING domain, and its SUMO ligase activity has been documented by several studies along with multiple substrates. The Nse1 kite subunit contains a variant RING (vRING) domain (C_4_HC_3_ instead of the canonical C_3_HC_4_ configuration of the zinc-coordinating residues) next to its WHB domain. Because RING domains often confer ubiquitin ligase activity by mediating interactions with the E2 ubiquitin-conjugating enzymes [15], Nse1 has been considered a likely ubiquitin ligase candidate. Initial studies of the Nse1 vRING domain in the fission yeast showed that the domain plays a structural role in the stabilization of the Nse1/3/4 subcomplex [6,12,16]. The vRING domain deletion and alanine mutations of the conserved cysteins (C197,199A) led to severe DNA damage sensitivity and decreased the presence of SMC5/6 at foci induced by DNA damage [16]. Later on, serine mutations in the conserved vRING domain cysteins (C199S, C216S) were found to suppress the DNA-damage sensitivity of various SMC5/6 mutants [17]. Though some studies were unable to detect ubiquitin-ligase activity of Nse1 using yeast or human proteins [16,17], Doyle et al. observed such activity of human NSE1 [18]. This study showed that NSE1 could work with UBC13/MMS2 as the E2 enzyme [18], and the NSE3 interaction of NSE1 was found to promote its E3 activity [18,19]. More recently, Weon et al. observed that in humans, where over 60 Nse3 paralogs called MAGE (Melanoma-associated anti-gene) are present, the NSE1 ubiquitin ligase can work independently of the SMC5/6 complex, form a complex with MAGE-F1, and ubiquitinate MMS19 in vivo to regulate the cytosolic iron-sulfur cluster assembly pathway [20]. However, no SMC5/6-associated-ubiquitination substrate has been identified so far, impeding our understanding of this SMC5/6-mediated function.

Here, we use *Schizosaccharomyces pombe* as a model organism to confirm that Nse1 is indeed able to promote ubiquitination. We demonstrate that not only the presence of Nse3 but also Nse4 promotes the Nse1 ligase activity. From the E2s tested, Nse1-dependent formation of ubiquitin conjugates can be observed only in the presence of Ubc13/Mms2, suggesting that it is the cognate E2 of Nse1. The connection between Nse1 and the ubiquitin pathway is further supported by the observation of an Nse1-ubiquitin interaction. We also identify the first ubiquitination-specific Nse1 mutation and demonstrate that it causes increased sensitivity to HU and MMS and synthetic phenotypes with *smc5/6* hypomorphic mutants. Importantly, by combining in vitro and in vivo approaches, we pinpoint Nse4 lysine K181 as the first known substrate of the SMC5/6-associated Nse1, reaching a milestone in the understanding of the SMC5/6-ubiquitination relationship.

## 2. Materials and Methods

### 2.1. Plasmids

Plasmids used for expression of *S. pombe* E1 (Uba1) and E2s (Ubc1, Ubc2, Ubc4, Ubc8, Ubc13) were described previously [21]. In the case of Mms2, its coding sequence (lacking introns) was synthesized by Genscript (Piscataway, NJ, USA) and inserted into *BamHI* and *SalI* sites of pGEX-6P-1 vector to create plasmid pPK14. Ubc7 sequence was amplified from *S. pombe* cDNA using primers oPK154 and oPK155 (Table S1) and inserted into the *NdeI* site of pET28b using In-fusion cloning kit (Takara, San Jose, CA, USA), creating plasmid pPK92. Plasmids used for the expression of Nse1/3 (KC600) and Nse1/3/4 (KC661) were described in [22]. For sole Nse1 expression, Nse1 was amplified using primers KB22 and KB178 (Table S1) and cloned into the *BamHI*-*SalI* sites of the pRSFDuet-1 vector, creating plasmid KB11-1.

In the case of the yeast two-hybrid constructs, the full-lenght *Nse1*(1–232) was inserted into *NdeI*-*EcoRI* sites of pGBKT7 (plasmid #10), the fragments of *Nse1*(1–116) and *Nse1*(117–232) were inserted into *NdeI*-*SalI* sites of pGBKT7 (plasmids KA45 and #619, respectively), and fragment *Nse1*(97–178) was amplified using primers MP011 and MP014 (Table S1) and inserted into *NcoI*-*BamHI* sites of pGBKT7 (plasmid CM013). The complete coding sequence of Nse4 was cloned into *NcoI*-*EcoRI* sites of pACT2 (plasmid #37), and the sequence of Ubiquitin was amplified using primers oPK586 and oPK588 and inserted into the *NdeI* site of pGADT7 (plasmid pPK296). 

In order to create a construct for *S. pombe* genome integration of Nse1 mutants, the genomic sequence of *Nse1* followed by *Ura4* gene and first 400 bp of *Nse1* 3′ UTR was inserted into *XhoI*-*PstI* sites of pBluescript II KS vector (plasmid SV16). To prepare the Nse1-HBH plasmid, the HBH sequence was amplified using primers oPK62 and oPK63 and cloned into the *NcoI* site (located near the stop codon of *Nse1*) of SV16 (plasmid pPK22). To create an integration construct for Nse4, its genomic sequence along with 139 bp of its 5′ UTR and 375 bp following the stop codon was amplified using primers oPK68 and oPK192 and inserted into pGEM-T Easy Vector. The KANMX6 sequence was then amplified by primers oPK195 and oPK196 and inserted into the *BglII* site (located in the *Nse4* 5′ UTR) of the Nse4-pGEM vector, creating plasmid pPK114. 

The point mutants of all constructs were prepared using the QuikChange Lightning Site-Directed Mutagenesis Kit (Agilent Technologies, Santa Clara, CA, USA) and primers described in Table S1.

### 2.2. Expression and Purification of Recombinant Proteins

The *S. pombe* E1 protein Uba1 and E2 enzymes (Ubc1, Ubc2, Ubc4, Ubc7, Ubc8, and Ubc13) were expressed in *E. coli* strain BL21 (DE3) RIL and purified similarly as described [21]. Cultures containing the Mms2-pGEX-6P-1 plasmid were grown at 37 °C to OD_600_ ~ 1 and induced by 1 mM IPTG at 37 °C for 3 h. The cell pellet was resuspended in buffer A containing 20 mM Tris-HCl (pH 8.0), 150 mM NaCl, 10% glycerol, 1 mM DTT, and protease inhibitors. The cells were lysed by sonication, centrifuged, and supernatant mixed with Glutathione Sepharose 4 Fast Flow resin (GE Healthcare, Chicago, IL, USA) at 4 °C for 1 h. Proteins were eluted by gravity using buffer A containing 10 mM glutathione. The GST tag was cleaved off using PreScission protease overnight at 4 °C. The protein mixture was then subjected to gel filtration on Superdex 75 (GE Healthcare) in buffer A. Fractions containing Mms2 were concentrated and snap-frozen in liquid nitrogen. The Nse1/3/4 trimer and its vRING mutant were prepared as described [22]. The *S. pombe* Nse1 protein, its mutants, and Nse1/3 dimer were expressed and purified similarly as in the case of human Nse1/3 dimer [22]. 

### 2.3. In Vitro Ubiquitination Assay

In vitro ubiquitination assays were performed in a 10 μL reaction volume containing 0.1 μM Uba1, 0.5–1 μM E2, 2.5–5 μM E3 (Nse1, Nse1/3, or Nse1/3/4), 2.5 μM biotinylated ubiquitin (Viva Bioscience, Exeter, UK), 0.5 mM ATP, 10 mM MgCl_2_, 50 mM Tris-HCl pH 7.5, and 50 mM NaCl. Reactions were incubated at 37 °C for 1 h, stopped by adding 10 μL of SDS Laemmli buffer (62.5 mM Tris–HCl, 2% SDS, 5% β-mercaptoethanol, 10% glycerol, 0.002% bromophenol blue) and subjected to 12% SDS-PAGE and Western blotting. Detection of ubiquitin conjugates was performed using Streptavidin-HRP (Vector Laboratories, Burlingame, CA, USA).

### 2.4. Yeast Two-Hybrid Assay

The *GAL4*-based yeast two-hybrid assay was used to analyze *S. pombe* interactions. The pGADT7 or pACT2 (carrying *GAL4* transcription activation domain) and pGBKT7 (carrying *GAL4* DNA-binding domain) plasmids with corresponding genes were co-transformed into the *S. cerevisiae* strain PJ69–4a and selected on plates lacking leucine and tryptophan. Strains were then grown to OD_600_ ~ 1 and fivefold serially diluted. Activation of the reporter genes was analyzed on media lacking leucine, tryptophan, and histidine (without or with 1 mM 3-aminotriazole) or adenine. Cells were grown for 3 days at 30 °C and scanned. 

### 2.5. Yeast Strains

Standard genetic techniques were used for the preparation of *S. pombe* strains [23]. Genotypes of the used strains are described in Table S2. The *nse1* and *nse4* mutant strains were prepared by transformation of the corresponding integration constructs digested by *XhoI* with *PstI* (*nse1*) or *NotI* (*nse4*) and selection on plates lacking uracil (*nse1*) or containing 0.1 mg/mL G418 (*nse4*).

To evaluate their sensitivity to DNA damaging agents, strains were grown to OD_600_ ~ 1, tenfold serially diluted, and spotted onto rich media (YES—0.5% yeast extract, 3% glucose, 225 mg/L amino acids) with indicated amounts of DNA damaging agents. Plates were then incubated at 28 °C for 3 days and scanned. 

### 2.6. Tandem Affinity Purification of Nse1-HBH

*S. pombe* Nse1 was C-terminally tagged by His-biotinylation signal-His (HBH) at its endogenous locus and purified as described [24]. Briefly, proteins were cross-linked by 1% formaldehyde to enable detection of transient interactions, Nse1-HBH-containing complexes were purified under denaturing conditions using Ni sepharose 6 Fast Flow (GE Healthcare) and Streptavidin agarose (Thermo Fisher Scientific, Waltham, MA, USA), digested by trypsin (Promega, Madison, WI, USA) and analyzed by mass spectrometry (MS). To identify dependence on Nse1 ligase activity, parallel purification of *nse1-R188E-HBH* ligase mutant was performed. An untagged strain was used to identify the contaminants.

### 2.7. Liquid Chromatography-Mass Spectrometry Analyses

Protein samples were digested using sequencing-grade trypsin (Promega). Liquid chromatography-mass spectrometry (LC-MS) analyses of peptide mixture were done using an RSLCnano system connected to an Orbitrap Elite hybrid spectrometer (Thermo Fisher Scientific). Prior to LC separation, peptide mixtures were online concentrated and desalted using a trapping column (100 μm × 30 mm) filled with 3.5-μm X-Bridge BEH 130 C18 sorbent (Waters, Milford, MA, USA). After washing of trapping column with 0.1% FA, the peptides were eluted (flow 300 nL/min) from the trapping column onto an Acclaim Pepmap100 C18 column (2 µm particles, 75 μm × 250 mm; Thermo Fisher Scientific) by approximately 1 or 2 h gradient program (mobile phase A: 0.1% FA in water; mobile phase B: 0.1% FA in 80% acetonitrile) with initial and end mobile phase B composition of 1% and 56%, respectively. Equilibration of the trapping column and the column was done prior to sample injection to the sample loop. The analytical column outlet was directly connected to the Nanospray Flex Ion Source (Thermo Fisher Scientific).

MS data were acquired in a data-dependent strategy selecting up to 10 precursors based on precursor abundance in the survey scan (350–2000 *m*/*z*). The resolution of the survey scan was 60,000 (400 *m*/*z*) with a target value of 1 × 10^6^ ions, 1 microscan, and a maximum injection time of 200 ms. HCD MS/MS spectra were acquired with a target value of 50,000 and resolution of 15,000 (400 *m*/*z*). The maximum injection time for MS/MS was 500 ms. Dynamic exclusion was enabled for 45 s after 1 MS/MS spectra acquisition, and early expiration was disabled. The isolation window for MS/MS fragmentation was set to 2 *m*/*z*. In the case of the 2nd and or 3rd analysis of the same sample solution, the scheduled precursor list (SPL) feature was used to exclude precursors already identified (false discovery rate 1%) in the 1st or 1st and 2nd analysis. Mass tolerance for *m*/*z* exclusion was set to 10 ppm and retention time window to 2 min. Please, see the raw data or exported LC and MS methods for more details (ProteomeXchange: PXD029573).

The analysis of the mass spectrometric RAW data files was carried out using the Proteome Discoverer software (Thermo Fisher Scientific; version 1.4) with in-house Mascot (Matrixscience, London, UK; version 2.4.1) and optionally also Sequest HT search engines utilization. MS/MS ion searches were done against the UniProt protein database for *Schizosaccharomyces pombe* or *Homo sapiens*. Please, see msf files or exported files covering the data processing pipeline used in Proteome Discoverer for more details (ProteomeXchange: PXD029573). The two or three analyses of the sample solution were searched together as a single data set.

### 2.8. Protein Modelling

The *S. pombe* Nse1 and Ubc13 structures were downloaded from AlphaFold protein structure database [25]. The Nse1(vRING)-Ubc13 and Nse1-Ubc13-Ubiquitin models, most similar to the 7BBD crystal structure of the TRIM21-Ubc13-Mms2-Ubiquitin complex [26], were created manually with the help of the COZOID tool [27].

## 3. Results

### 3.1. Nse1 Is a Ubiquitin Ligase

**Figure 1:**
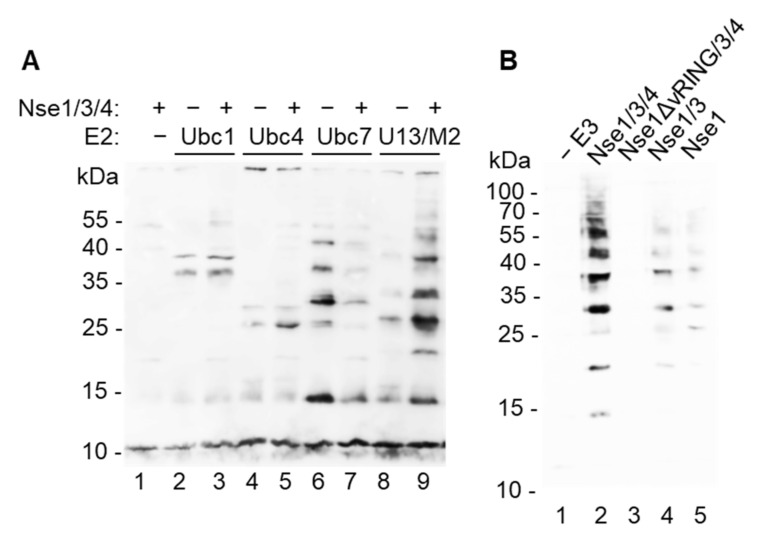
*S. pombe* Nse1 promotes in vitro ubiquitination together with Ubc13/Mms2. (**A**) Nse1 stimulates ubiquitin-chain formation when combined with Ubc13/Mms2. *S. pombe* E1 (Uba1), indicated E2s, biotinylated ubiquitin, ATP, and MgCl_2_ were incubated in the presence or absence of Nse1/3/4 trimer for 1 h at 37 °C. The mixture was separated on 12% SDS–PAGE followed by Western blotting and visualization of biotinylated ubiquitin using Streptavidin-HRP. Numbers on the left indicate molecular weights of protein standards (in kDa). (**B**) Nse1/3/4 trimer promotes ubiquitination more efficiently than Nse1/3 dimer or Nse1 alone, and deletion of its vRING domain ablates this activity. In vitro ubiquitination assay was performed using E1, Ubc13/Mms2, biotinylated ubiquitin, indicated E3, and analyzed as in (**A**).

Though Nse1 contains a variant RING domain characteristic of ubiquitin ligases and human NSE1 has been shown to possess E3 ubiquitin-ligase activity in vitro [18], other studies using *S. pombe* and human proteins were unable to show this activity [16,17]. To understand this discrepancy, we purified fission yeast E1 ubiquitin-activating enzyme (Uba1), E2 conjugating enzymes (Ubc1, Ubc2, Ubc4, Ubc7, Ubc8, Ubc13/Mms2), and Nse1 either on its own, in dimer with Nse3, or in trimer with Nse3 and Nse4 ([22], Figure S1), and analyzed the Nse1 ligase activity. When the proteins were combined together with biotinylated ubiquitin, we observed significant ubiquitination stimulation only in the presence of Ubc13/Mms2 (Figure 1A and Figure S2A), suggesting that this E2 enzyme is specifically working with Nse1. The Nse1/3/4 trimer showed increased formation of ubiquitin conjugates when compared to Nse1 on its own or in dimer with Nse3 (Figure 1B), indicating that Nse1 needs to be in a proper conformation or a composite surface of the Nse1/3/4 subcomplex is required for the proper activity. As expected, deletion of the Nse1 vRING domain disrupted the ligase activity (Figure 1B). Omission of E1, Ubc13, ubiquitin or ATP also led to complete disruption of ubiquitin conjugate formation (Figure S2B). Together these results confirm the Nse1 ubiquitin ligase activity and show its specific association with Ubc13/Mms2.

### 3.2. Nse1 Ligase Activity Is Abolished by the Nse1-R188E Mutation

**Figure 2:**
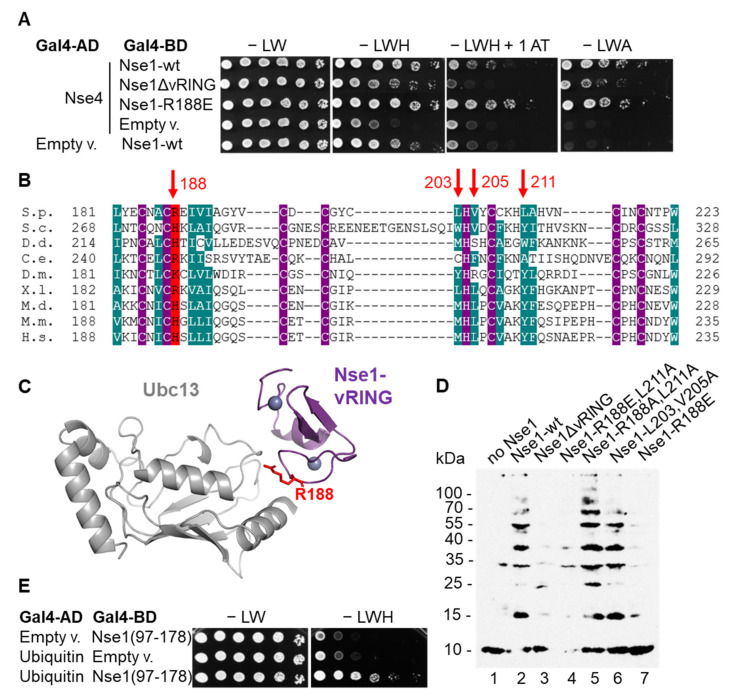
Nse1 mutation R188E disrupts its ubiquitin ligase activity. (**A**) Nse1–Nse4 interaction analyzed by yeast two-hybrid assay is decreased by Nse1 vRING removal, but not by its R188E mutation. Plasmids carrying indicated genes or corresponding empty vectors were co-transformed into the *S. cerevisiae* PJ69–4a strain, grown to OD_600_ ~ 1, fivefold serially diluted and spotted on solid media lacking leucine (L), tryptophan (W), histidine (H), or adenine (A) in absence or presence of 1 mM 3-aminotriazole (1AT), as depicted. Cells were grown for 3 days at 30 °C and scanned. (**B**) Alignment of the conserved vRING domain sequences of Nse1 from different species: *S. pombe* (S.p.), *S. cerevisiae* (S.c.), *Dictyostelium discoideum* (D.d.), *Caenorhabditis elegans* (C.e.), *Drosophila melanogaster* (D.m.), *Xenopus laevis* (X.l.), *Monodelphis domestica* (M.d.), *Mus musculus* (M.m.), *Homo sapiens sapiens* (H.s.). Amino acid shading represents the following conserved amino acids: violet—Zn^2+^-coordinating cysteins and histidines; dark green—hydrophobic and aromatic; red—basic. Red arrows on top indicate amino acids mutated in (**D**) and their corresponding numbers in *S. pombe*. (**C**) The Nse1 vRING domain (dark violet)—Ubc13 (grey) interaction model based on the crystal structure of the human TRIM21-Ubc13 [26]. The Nse1-R188 residue is red labeled. (**D**) Nse1 R188E mutation impairs its ability to promote ubiquitination in vitro. E1, Ubc13/Mms2, biotinylated ubiquitin, and indicated Nse1 variants were incubated with ATP and MgCl_2_ for 1 h at 37 °C and analyzed as in Figure 1A. (**E**) Nse1(97–178) interacts with ubiquitin in the yeast two-hybrid assay. *S. cerevisiae* PJ69–4a transformants were analyzed as in (**A**).

Though deletion of the Nse1 vRING domain disrupted its ligase activity (Figure 1B), such deletion likely disrupts the overall structure of the Nse1/3/4 trimer [10,12,16]. In agreement, we observed a decrease in Nse1–Nse4 interaction in the yeast-two-hybrid assay when the vRING domain was deleted (Figure 2A). Therefore, we wished to identify a milder mutation that would specifically abolish the E3 ligase activity but would not otherwise impede the Nse1/3/4 stability. We used the fact that E3 ligases usually work by stimulating the interaction between E2s and the substrate and tested several mutations of the conserved amino acids of the Nse1 vRING surface supposedly interacting with E2 ([18], Figure 2B,C). Among these mutants, Nse1-R188E, corresponding to human H195 (Figure 2B), led to a significant decrease in Nse1 ligase activity (Figure 2D). At the same time, it did not affect the Nse1–Nse4 interaction (Figure 2A); therefore, it is the first yeast mutant that selectively disrupts the Nse1 ubiquitin ligase activity (see below).

### 3.3. Nse1 Associates with Ubc13/Mms2 and Ubiquitin

To better understand the mechanism of Nse1 function as a ubiquitin ligase, we tested Nse1 interactions with ubiquitination machinery proteins. We tagged Nse1 at the endogenous locus by His and biotinylation signal (HBH-tag) and first confirmed that it did not affect yeast cell growth or DNA-damage sensitivity (Figure S3). We then stabilized transient interactions within the yeast cells by formaldehyde cross-linking, purified the Nse1-containing complexes, and identified proteins by MS. The untagged strain was used to subtract the background proteins. Among the Nse1-bound factors, we repeatedly observed the Ubc13, Mms2, and Uba1 (Data File S1A,B, Data File S2A–C—ProteomeXchange: PXD029573, and Table S3). Importantly, they were present in the Nse1-HBH complexes but not in its ligase mutant R188E, confirming the notion that Nse1 interacts with Ubc13/Mms2 and R188E eliminates this interaction. The E1 interaction is likely only mediated by Ubc13/Mms2. Further, using the yeast two-hybrid assay, we discovered that Nse1 interacts with ubiquitin itself, and the interaction site is located in the WHB (aa 117–178) domain of Nse1 (Figure 2E and Figure S4). These data strengthen the connection between Nse1 and the ubiquitination pathway and again show a specific role of Ubc13/Mms2.

### 3.4. Nse1 E3 Activity Plays an Important Role during Replication Stress

**Figure 3:**
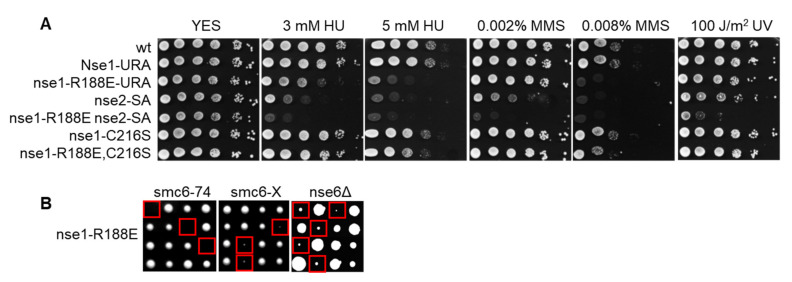
*Nse1-R188E* ubiquitin ligase mutant shows increased sensitivity to HU, MMS, and synthetic phenotypes with *smc5/6* mutants. (**A**) *Nse1-R188E* strain is sensitive to HU and MMS. The indicated *S. pombe* strains were grown to OD_600_ ~ 1, tenfold serially diluted, spotted onto rich media with the designated amounts of HU, MMS, or UV dose, and incubated at 28 °C for 3 days. (**B**) *Nse1-R188E* mutant shows synthetic lethality with *smc6-74* and severe growth defects with *smc6-X* and *nse6Δ*. Double mutants were analyzed by tetrad dissection of *S. pombe* diploid strains resulting from crosses between the strain carrying the *nse1-R188E* mutation and the indicated *smc5/6* mutants. Red rectangles mark double mutants.

To evaluate the importance of the Nse1 ligase activity for the SMC5/6 function in vivo, we examined the phenotype of the fission yeast *nse1*-*R188E* ligase mutant. We observed its increased sensitivity to HU and MMS (Figure 3A). The fact that the HU sensitivity of the mutant was especially pronounced suggests that Nse1 ligase activity is particularly important when cells need to cope with replication stress. The Nse1 ubiquitin ligase mutant showed a synthetic relationship with the Nse2 SUMO ligase mutant (C195S, H197A), indicating their separate roles in SMC5/6 function. Interestingly, the addition of *nse1*-*C216S* mutation suppressed the R188E phenotypes (Figure 3A), suggesting that it leads to a ubiquitin-ligase-independent outcome. When *nse1-R188E* was combined with other *smc5/6* mutations, we observed lethality in the case of *smc6-74* (A151T) and severe growth defects with *smc6-X* (R706C) and *nse6Δ* (Figure 3B). These synthetic phenotypes were again suppressed by the *nse1-C216S* mutation (Figure S5). Together these data show that the ubiquitin ligase role of Nse1 is particularly important under replication stress, and its absence aggravates the defects of other *smc5/6* mutants.

### 3.5. Nse4 and Nse3 Are Substrates of Nse1 Ubiquitin Ligase

Next, we wished to identify the first substrates of the SMC5/6-associated Nse1 ubiquitin-ligase. We considered SMC5/6 subunits as the most likely candidates, similarly as in the case of Nse2 SUMO ligase [28]. Therefore, we analyzed the above-described in vitro ubiquitination assay mixture (Figure 1) containing the Nse1/3/4 subcomplex together with E1, Ubc13/Mms2, and ubiquitin by MS. Interestingly, we observed ubiquitination of Nse4 at lysine K181 and Nse3 at lysine K195 (Data File S3 and Data File S4). To evaluate their modifications in vivo, we analyzed ubiquitination of the Nse1-containing complexes by MS, using the above-described *Nse1-HBH* purification. The analysis confirmed Nse4 ubiquitination at K181 (Data File S1A), but we were unable to detect ubiquitination of Nse3. The fact that ubiquitination of Nse4 was absent in the *nse1-R188E-HBH* mutant strain (Data File S1B) verifies its dependence on Nse1.

**Figure 4:**
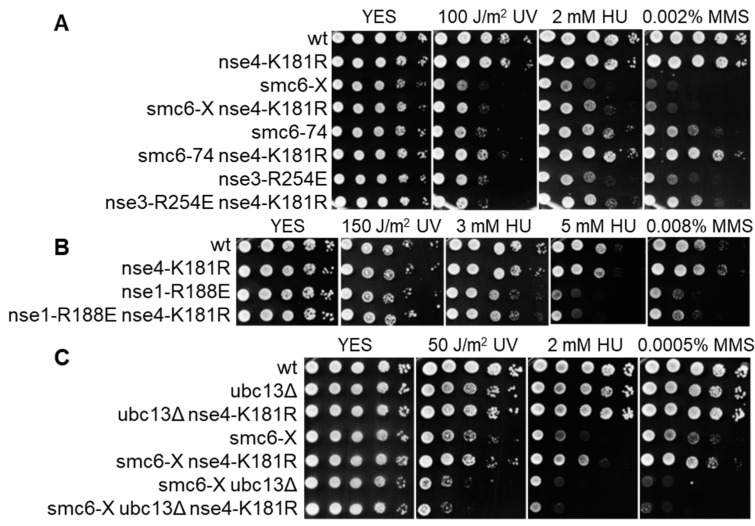
*Nse4-K181R* mutation partially suppresses the sensitivities of *smc6-X*, *smc6-74*, and *nse3-R254E* to DNA-damaging agents and HU. (**A**) Sensitivity of depicted *smc5/6* mutants to UV, HU, and MMS is partially suppressed by *nse4-K181R*. (**B**) *Nse4-K181R* does not suppress the sensitivity of the *nse1-R188E* strain. (**C**) Suppression of *smc6-X* sensitivity by *nse4-K181R* is dependent on *Ubc13*. The indicated *S. pombe* strains were grown to OD_600_ ~ 1, tenfold serially diluted, spotted onto rich media with specified amounts of DNA-damaging agents, and incubated at 28 °C for 3 days.

To assess the importance of Nse4 modification at K181 in vivo, we introduced its mutation to arginine, which cannot be ubiquitinated but retains the positive charge. This mutation did not lead to increased DNA damage sensitivity (Figure 4A). Interestingly, when combined with other *smc5/6* mutants (*smc6-74*, *smc6-X*, *nse3-R254E*), it partially suppressed their sensitivity to DNA-damaging agents and HU (Figure 4A). However, *nse4-K181R* was not able to suppress *nse6Δ* (Figure S6A). *Nse4-K181R* also did not suppress the sensitivity of *nse1-R188E*, which is in line with its dependence on the Nse1 ligase activity (Figure 4B). In agreement with the above results, the sensitivity of *smc6-X* was also not suppressed by *nse4-K181R* in the absence of Ubc13, showing that Ubc13 plays an important role in Nse1-dependent Nse4 ubiquitination (Figure 4C). This effect is *ubc13Δ*-specific, as deletion of other E2 (*ubc7Δ* in Figure S6B) does not abolish the suppression. In summary, the above-described data identify Nse4 as the first known SMC5/6-associated Nse1 ubiquitination substrate and support the role of Ubc13 in this process.

## 4. Discussion

### 4.1. Nse1 Is a Ubiquitin Ligase Working Specifically with Ubc13/Mms2

Because SMC5/6 subunit Nse1 contains a variant RING domain, it has long been considered a possible ubiquitin ligase. However, in contrast to the SUMO ligase function (conferred by Nse2), the ubiquitin ligase activity of SMC5/6 still remains a mystery. Until now, only two studies observed ubiquitin ligase activity of human and mouse NSE1 in vitro [18,29], whereas other studies observed no such activity using human or *S. pombe* proteins [16,17]. Here, we purified corresponding proteins from the fission yeast and showed that Nse1 is indeed able to promote ubiquitination in vitro, supporting the conservation of this activity among species. Doyle et al. observed human NSE1 activity stimulation by NSE3 [18], and we were able to purify not only Nse1/3 dimer and confirm Nse3 stimulation, but also the entire yeast Nse1/3/4 subcomplex and show it possesses the highest ligase activity (Figure 1B). We assume that the fact that some studies did not observe the ubiquitin ligase activity of Nse1 can be explained by its relatively low efficiency and the dependence of its function on Nse3 and Nse4.

Doyle et al. also observed that the human NSE1/3 was active together with UBC13/MMS2 [18]; however, because the absence of this activity with other E2s was not shown, its specificity to UBC13/MMS2 remained unclear. Here we tested six yeast ubiquitin-conjugating enzymes (Ubc1, Ubc2, Ubc4, Ubc7, Ubc8, Ubc13/Mms2) and showed that Nse1 indeed stimulates the formation of ubiquitin conjugates specifically in the presence of Ubc13/Mms2 (Figure 1A and Figure S2A). In contrast to these results, Hou et al. used four E2s (UBE2D2, D3, L3, k) combined with mouse NSE1 alone and observed the strongest stimulation with UBE2D2 [29], the orthologue of *S.p.*Ubc4. We consider this study less reliable because no other laboratory observed such strong ubiquitination stimulation using NSE1 alone, and due to the fact that this study reported the same E2 specificity for all three E3 ubiquitin ligases tested [29].

Compared to the previous studies, we provide further data supporting the relationship between Nse1 and Ubc13/Mms2. First, we identified Ubc13 and Mms2 among the Nse1-HBH precipitated factors, absent when the ubiquitin ligase mutant *nse1-R188E-HBH* was used (Data File S1A,B and Data File S2A–C). Note that the Nse1-precipitated proteins also included Ubc4 and Ubc7, with Ubc7 also being absent in *nse1-R188E-HBH*. However, the parallel presence of Ubc4 in the untagged-strain precipitates prevents drawing conclusions about its specific association with Nse1. Second, our genetic analysis showed the loss of *nse4-K181R*-dependent suppression of *smc6-X* in the absence of Ubc13 (Figure 4C), indicating that Nse1-dependent ubiquitination of Nse4 utilized Ubc13/Mms2. In contrast, deletion of Ubc7 did not affect *nse4-K181R*-dependent suppression of *smc6-X* (Figure S6B), further strengthening our conclusion on the specific interplay between Nse1 and Ubc13/Mms2.

The fact that we did not observe Nse1 ubiquitin ligase activity in vitro with an E2 other than Ubc13/Mms2, however, does not exclude the possibility that Nse1 also utilizes another E2 (e.g., Ubc7) for its function. The described in vitro ubiquitination assay generally relies on ubiquitin itself as the substrate for modification (leading to ubiquitin chain formation). Therefore, Nse1-dependent monoubiquitination would not be observable in the absence of a natural substrate protein. Likewise, chain initiation and elongation are often carried out by separate E2s, and Ubc13/Mms2 has been shown to produce K63 ubiquitin chains after previous lysine monoubiquitination by other E2s [15,30,31]. Therefore, it is possible that Nse1 needs another E2 to first monoubiquitinate the substrate before it can stimulate its polyubiquitination with Ubc13/Mms2.

**Figure 5:**
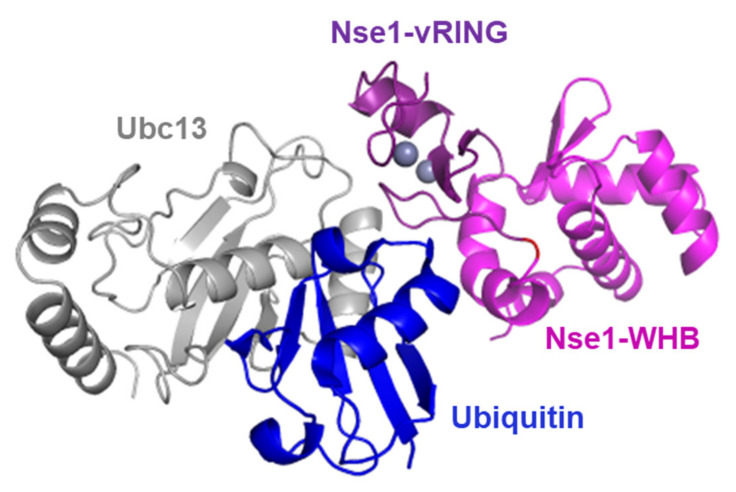
Model for Nse1–Ubc13~Ub complex. The Ubc13 E2 enzyme (grey) is charged with ubiquitin (blue) and binds the Nse1-vRING domain (dark violet). Ubiquitin is localized in the vicinity of the Nse1-WHB domain (light violet), and their interaction may enhance the ubiquitination process.

The fact that Nse1 directly interacts with ubiquitin (Figure 2E) further strengthens its connection to the ubiquitination pathway and may explain the mechanism Nse1 uses to promote ubiquitin chain formation. The RING-domain E3 ligases have been shown to bind both the E2 and its covalently-bound ubiquitin to lock them in a conformation primed for catalysis and stimulate ubiquitination [26,32,33,34]. Therefore, based on published structures of a RING-Ubc13~Ub/Mms2 complex [26] and Nse1/3/4 [13], we modeled the Nse1–Ubc13~Ub complex (Figure 5). Accordingly, the ubiquitin that is bound to Ubc13 by a thioester bond is localized in the vicinity of the WHB domain of Nse1, which we found to be the domain mediating the Nse1-ubiquitin interaction (Figure 2E). We hypothesize that the Ubi-WHB interaction may enhance the ubiquitination reactions.

### 4.2. Nse1 Mutation R188E Specifically Impairs Its Ubiquitin Ligase Function

Until this study, the importance of Nse1 ubiquitin ligase activity remained unclear because of the structural role that its vRING domain plays in the SMC5/6 complex ([12,16] and Figure 2A), which made it difficult to specifically impair the ligase function. Here, we sought to identify a specific mutation and targeted conserved Nse1 amino acids potentially interacting with the E2 (Figure 2B,C). Among these, Nse1-R188E showed to be the most promising as it substantially decreased the Nse1 ubiquitin ligase activity in vitro (Figure 2D) but seemed not to confer a structural defect (as observed for the Nse1–Nse4 interaction—Figure 2A). The importance of R188 for the E2 interaction in vivo was confirmed during the Nse1 tandem affinity purification as its mutation led to the loss of Ubc13/Mms2 coprecipitation (Data File S1A,B and Data File S2A–C). To establish the importance of the Nse1 ubiquitin ligase activity, we introduced the R188E into yeast and observed its increased sensitivity, particularly to replication stress induced by HU (Figure 3A). The ubiquitin ligase function is not essential, consistent with previous results using the deletion of the whole vRING domain [16,17]. However, the R188E mutation causes synthetic lethality or severe growth defects when combined with other *smc5/6* mutants (Figure 3B).

Our *nse1-R188E* ubiquitin ligase mutant also showed a synthetic relationship with the Nse2 SUMO ligase mutant (C195S, H197A), indicating their separate roles in the SMC5/6 function. The observation that the previously described vRING mutation C216S [17] suppressed the R188E mutant (Figure 3A) implies its ubiquitin-ligase-independent role. This notion was also confirmed in the in vitro ubiquitination assay, where C216S did not lead to a decrease in ubiquitin conjugate formation (Figure S7). These results again point to the multitude of functions the vRING domain plays in the SMC5/6 regulation.

### 4.3. Nse1 Ligase Substrates

After confirming the Nse1 ubiquitin ligase activity and evaluating its importance, we wished to determine the first SMC5/6-associated-ubiquitination substrate. The MS analysis of the Nse1/3/4- and Ubc13/Mms2-containing in vitro ubiquitination assay led to the identification of Nse4 ubiquitination at K181 and Nse3 at K195 (Data File S3 and Data File S4). Evaluation of the Nse1-HBH and Nse1-R188E-HBH precipitates confirmed Nse4 ubiquitination in vivo in an Nse1-dependent manner (Data File S1A,B). The fact that we did not detect Nse3 ubiquitination in yeast does not exclude this possibility, as modified peptides usually constitute only a small fraction of all peptides. Importantly, the identification of Nse1-dependent ubiquitination of yeast Nse4 and Nse3 is in agreement with our previous results using their human counterparts [19]. The observation that the established human NSE4B ubiquitination takes place at K219 and K234 in HEK293T cells (Data File S5, acquired but not published in [19]) indicates the conservation of this process, as the modified sites both in human and yeast are located exactly before the C-terminal domain of Nse4 (known to interact with the SMC5 cap, [7]). Furthermore, we observed ubiquitination of the human NSE3 orthologue (MAGEG1) at K220 and K221 [19]. Though we believe both Nse4 and Nse3 are substrates of the Nse1 ubiquitin ligase, the support of the yeast in vivo data makes Nse4 a stronger candidate, and we next concentrated on its genetic analysis.

Surprisingly, when we disrupted Nse4 ubiquitination in yeast by the *nse4-K181R* mutation, it showed the opposite genetic interactions to the *nse1-R188E* ligase mutant and partially suppressed the DNA-damage sensitivity of *smc6-X*, *smc6-74*, and *nse3-R254E* (Figure 4A). However, this suppression still appears to be dependent on the Nse1 ubiquitin ligase activity and its associated E2 Ubc13/Mms2, as *nse4-K181R* does not suppress *nse1-R188E* (Figure 4B) and the suppression of *smc6-X* is dependent on the presence of Ubc13 (Figure 4C). Therefore, it seems Nse1 plays a multifaceted role where ubiquitination of different substrates leads to varied outcomes. Identification of Nse4 as the first SMC5/6-associated Nse1 substrate represents an important step on the path to a better understanding of its ubiquitin ligase function.

## 5. Conclusions

In conclusion, this study clearly shows that the Nse1 subunit of the SMC5/6 complex carries a ubiquitin ligase activity, which is promoted by its binding partners Nse3 and Nse4 and utilizes Ubc13/Mms2 as the E2. We identify the first Nse1 ubiquitination-specific mutation (R188E) and show that the Nse1 ligase activity is particularly important under replication stress. The combination of in vitro and in vivo results pinpoint Nse4 (lysine K181) as the first known SMC5/6-associated Nse1 ubiquitination substrate.

## Data Availability

The mass spectrometry proteomics data have been deposited to the ProteomeXchange Consortium via the PRIDE [35] partner repository with the dataset identifier PXD029573.

## Supplementary Materials

The following are available online at https://www.mdpi.com/2073-4409/11/1/165/s1, Table S1: Oligonucleotides used in this study, Table S2: *S. pombe* strains used in this study, Table S3: Proteins identified by Mass Spectrometry, Figure S1: Proteins purified in this study, Figure S2: Nse1 promotes in vitro ubiquitination specifically with Ubc13/Mms2, Figure S3: The HBH tag of the *Nse1-HBH* strain does not affect yeast cell growth or DNA-damage sensitivity, Figure S4: The C-terminal part of Nse1 interacts with ubiquitin in the yeast two-hybrid assay, Figure S5: The synthetic lethality and growth defect observed in the *smc6-74 nse1-R188E* and *smc6-X nse1-R188E* strains, respectively, are suppressed by the *nse1-C216S* mutation, Figure S6: *Nse4-K181R* does not suppress the sensitivity of *nse6Δ*, but suppresses *smc6-X* in the absence of *Ubc7*, Figure S7: The C216S mutation does not inhibit Nse1 ubiquitin ligase activity.
