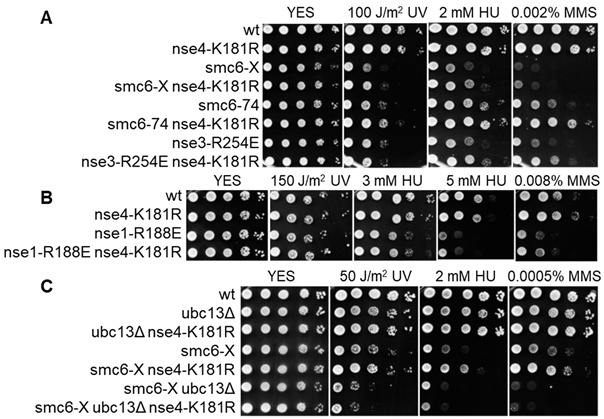# Correction: Kolesar et al. Role of Nse1 Subunit of SMC5/6 Complex as a Ubiquitin Ligase. *Cells* 2022, *11*, 165

**DOI:** 10.3390/cells13100812

**Published:** 2024-05-10

**Authors:** Peter Kolesar, Karel Stejskal, David Potesil, Johanne M. Murray, Jan J. Palecek

**Affiliations:** 1 National Centre for Biomolecular Research, Faculty of Science, Masaryk University, 62500 Brno, Czech Republic; 2 Central European Institute of Technology, Masaryk University, 62500 Brno, Czech Republic; karel.stejskal@imba.oeaw.ac.at (K.S.); david.potesil@ceitec.muni.cz (D.P.); 3 Genome Damage and Stability Centre, School of Life Sciences, University of Sussex, Falmer, Brighton BN1 9RH, UK; j.m.murray@sussex.ac.uk

## Error in Figure

In the original publication [1], there was a mistake in Figure 4 as published. An incorrect plate scan (0.002% MMS plate, taken after 5 days of growth) was placed in the 0.001% MMS panel in Figure 4A. The panel is excessive, and should not have been included in the publication. The corrected Figure 4 appears below. The authors state that the scientific conclusions are unaffected. This correction was approved by the Academic Editor. The original publication has also been updated.