# Supplementary material for: Role of Nse1 Subunit of SMC5/6 Complex as a Ubiquitin Ligase
Source: Cells. 2022 Jan 4;11(1):165. doi: 10.3390/cells11010165 (PMC8750328; doi:10.3390/cells11010165)
Supplement: Supplementary file 1 [file cells-11-00165-s001.zip › cells-1477579-supplementary/Supplementary Information.pdf]

Supplementary Information

Role of Nse1 subunit of SMC5/6 complex as a ubiquitin ligase

Peter Kolesar, Karel Stejskal, David Potesil, Johanne M. Murray and Jan J. Palecek

Table S1. Oligonucleotides used in this study.

oligonucleotide		sequence 5' to 3'
oPK154	Ubc7-F	cgcgcggcagccatatgagtaaagctatggcgttgc
oPK155	Ubc7-R	gtcatgctagccatattataatcccaaggttttacgagc
KB22	Nse1-R	gaggagaagcccggtgtcgacttaccagcgtcctataacggtcgc
KB178	Nse1-F	agccaggatccgggcgaaaacctgtattttcagggcatggagaaagagagacaag
MP011	NSE1-97-F	ctgcatatggccatgggtgttcagatagaacttatgagaaagataatcga
MP014	NSE1-178-R	gcaggtcgacggatccttactcgtattcgttgtgtaaataagcat
oPK586	Ub-F	cagattacgctcatatgcaaattttcgtcaaaaccc
oPK588	Ub-R	gcctccatggccatatcaaccaccacgaagacg
oPK62	Nse1-HBH-F	taacacgccatgggcaaatgcgaccgttataggacgctggcggatccccgggttaattaa
oPK63	Nse1-HBH-R	aaacaagtaaagcgaacaccagtaggtagtgaaaaaattagaattcgagctcgtttaaac
oPK68	Nse4-139-F	ggagagttgtgcttaaaggttg
oPK192	Nse4+375-R	taatctaatcatcctccgttgc
oPK195	G418-F	taacaagaagagatcagcttgcctcgtccccg
oPK196	G418-R	ttattgaatcagatctactggatggcggcgttag
oPK30	Nse1-C184*-F	cgaatacgagagcaatttatacgaatagaacgcttgtcgcga
oPK31	Nse1-C184*-R	tcgcgacaagcgttctattcgtataaattgctctcgtattcg
oPK32	Nse1-L203,V205A-F	gctaaatgcttgcaacagtaagcatgcgcgcagtagccacaatcacatac
oPK33	Nse1-L203,V205A-R	gtatgtgattgtggctactgcgcgcatgcttactgttgcaagcatttagc
oPK44	Nse1-R188A-F	caatttatacgaatgcaacgcttgtgccgaaattgtaattgctgga
oPK45	Nse1-R188A-R	tccagcaattacaatttcggcacaagcgttgcattcgtataaattg
oPK46	Nse1-R188E-F	caatttatacgaatgcaacgcttgtgaggaaattgtaattgctgga
oPK47	Nse1-R188E-R	tccagcaattacaatttcctcacaagcgttgcattcgtataaattg
oPK48	Nse1-L211A-F	ctgcttgcatgtttactgttgcaagcatgcagctcatgttaattgtataa
oPK49	Nse1-L211A-R	ttatacaattaacatgagctgcatgcttgcaacagtaaacatgcaagcag
JP903	Nse1–C216S-F	gcatttagctcatgttaatagtataaattgtaacacgcc
JP904	Nse1–C216S-R	ggcgtgttacaatttatactattaacatgagctaaatgc
oPK92	Nse4-K181R-F	cccaagaaaataacaccactagaaatgtcttgcacatatctcg
oPK93	Nse4-K181R-R	cgagatatgtgcaagacatttctagtggtgttattttcttggg

Table S2. *S. pombe* strains used in this study.

**strain #**	**genotype**	**source**
AMC503	*ade6-704 leu1-32 ura4-D18 h+*	A. M. Carr
yPK2	*nse1-HBH::Ura4 ade6-704 leu1-32 ura4-D18 h+*	This study
YJP101	*nse1-WT::Ura4 ade6-704 leu1-32 ura4-D18 h-*	This study
yPK1	*nse1-R188E::Ura4 ade6-704 leu1-32 ura4-D18 h+*	This study
JMM2050	*nse2-SA::Ura4 ade6-704 leu1-32 ura4-D18 h-*	J. M. Murray
yPK60-1B	*nse2-SA::Ura4 nse1-R188E::Ura4 ade6-704 leu1-32 ura4-D18 h+*	This study
YHS45	*nse1-C216S::Ura4 ade6-704 leu1-32 ura4-D18 h-*	This study
yPK180	*nse1-R188E C216S::Ura4 ade6-704 leu1-32 ura4-D18 h-*	This study
JMM956	*smc6-74 ade6-704 leu1-32 ura4-D18 h+*	J. M. Murray
JMM1920	*smc6-X ade6-704 leu1-32 ura4-D18 h-*	J. M. Murray
NBY835	*nse6::kanMX6 leu1-32 ura4-D18 h+*	M. N. Boddy
yPK208	*nse4-K181R-KanMX6 ade6-704 leu1-32 ura4-D18 h+*	This study
yPK213-2C	*smc6-X nse4-K181R-KanMX6 ade6-704 leu1-32 ura4-D18 h+*	This study
yPK229-11B	*smc6-74 nse4-K181R-KanMX6 ade6-704 leu1-32 ura4-D18 h-*	This study
MA23	*nse3-R254E::Lox ade6-704 leu1-32 ura4-D18 h+*	K. Zabrady
yPK228-5D	*nse3-R254E::Lox nse4-K181R-KanMX6 ade6-704 leu1-32 ura4-D18 h-*	This study
yPK231-3C	*nse1-R188E::Ura4 nse4-K181R-KanMX6 ade6-704 leu1-32 ura4-D18 h+*	This study
yPK200	*ubc13::KanMX6 ade6-704 leu1-32 ura4-D18 h+*	Bioneer library
yPK291-2A	*nse4-K181R-KanMX6 ubc13::KanMX6 ade6-704 leu1-32 ura4-D18 h+*	This study
yPK219-3C	*smc6-X ubc13::KanMX6 ade6-704 leu1-32 ura4-D18 h+*	This study
yPK242-9B	*smc6-X ubc13::KanMX6 nse4-K181R-KanMX6 ade6-704 leu1-32 ura4-D18 h+*	This study
yPK238-4C	*nse6::kanMX6 nse4-K181R-KanMX6 ade6-704 leu1-32 ura4-D18 h+*	This study
yPK43	*ubc7::kanMX6 ade6-704 leu1-32 ura4-D18 h+*	Bioneer library
yPK292-6C	*nse4-K181R-KanMX6 ubc7::kanMX6 ade6-704 leu1-32 ura4-D18 h+*	This study
yPK292-1B	*smc6-X ubc7::kanMX6 ade6-704 leu1-32 ura4-D18 h+*	This study
yPK292-6A	*smc6-X nse4-K181R-KanMX6 ubc7::kanMX6 ade6-704 leu1-32 ura4-D18 h+*	This study

Supplementary Figures:

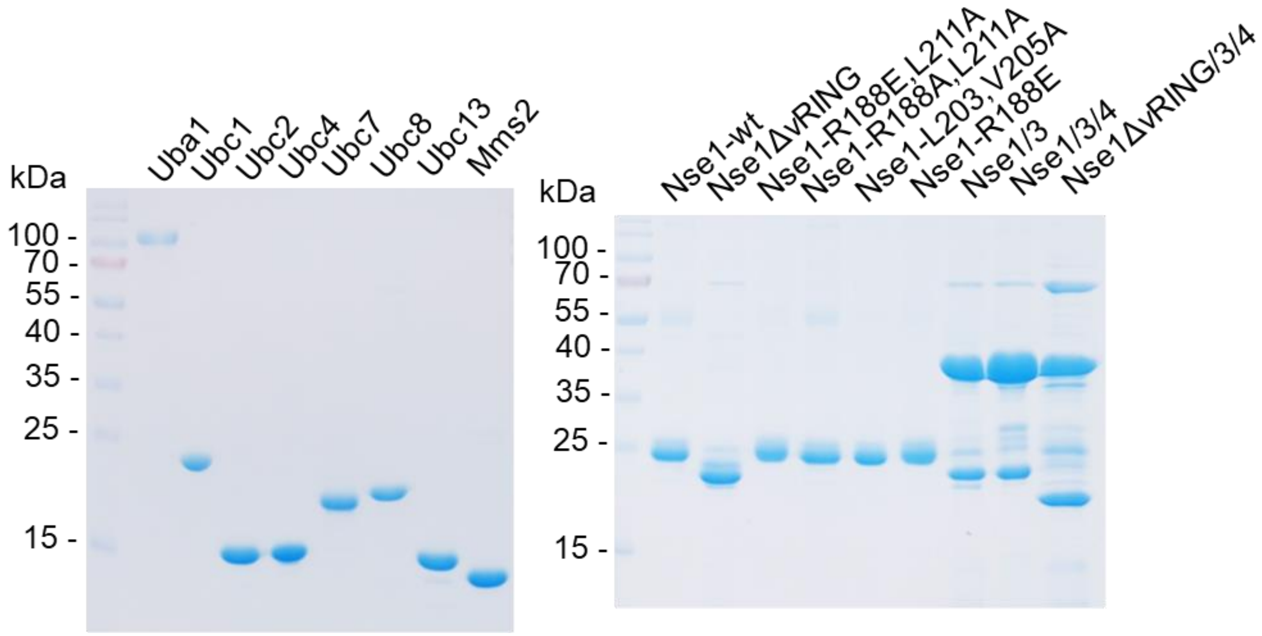

Figure S1. Proteins purified in this study. Indicated proteins were resolved by 12% SDS-PAGE and stained with Coomassie blue dye. Numbers on the left indicate molecular weights of protein standards (in kDa).

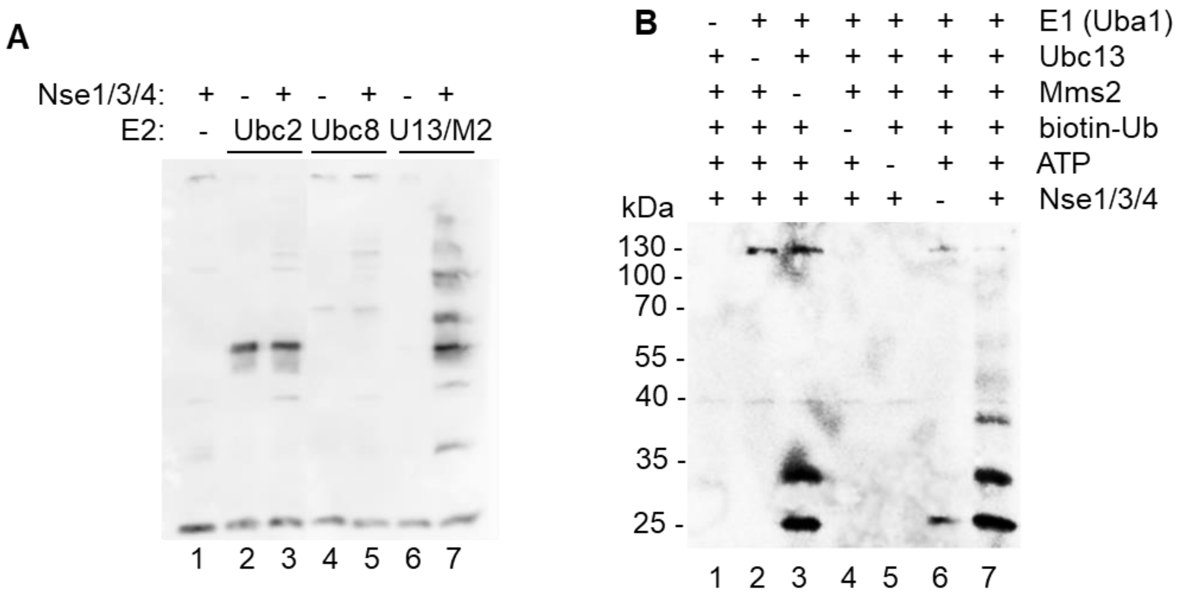

Figure S2. Nse1 promotes in vitro ubiquitination specifically with Ubc13/Mms2. (**A**) Nse1 does not stimulate ubiquitin chain formation when combined with Ubc2 or Ubc8. *S. pombe* E1 (Uba1), indicated E2s, biotinylated ubiquitin, ATP, and $MgCl_2$ were incubated in the presence or absence of Nse1/3/4 trimer for 1h at 37°C. The mixture was analyzed by 12% SDS–PAGE followed by western-botting and visualization of biotinylated ubiquitin using Streptavidin-HRP. (**B**) Omission of E1, Ubc13, ubiquitin or ATP disrupts ubiquitin conjugate formation. The indicated proteins were mixed and analyzed as in (A).

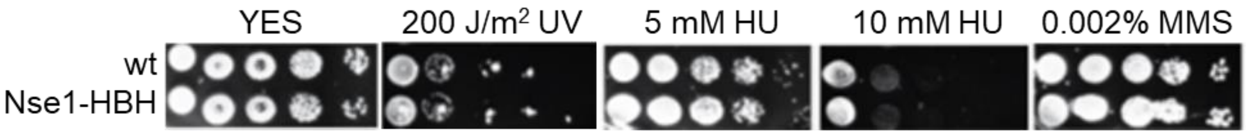

Figure S3. The HBH tag of the *Nse1-HBH* strain does not affect yeast cell growth or DNA-damage sensitivity. The *wild-type* and *Nse1-HBH* strains were grown to OD_{600} ~ 1, tenfold serially diluted, spotted onto rich media with the indicated amounts of HU, MMS or UV dose, and incubated at 28 °C for 3 days.

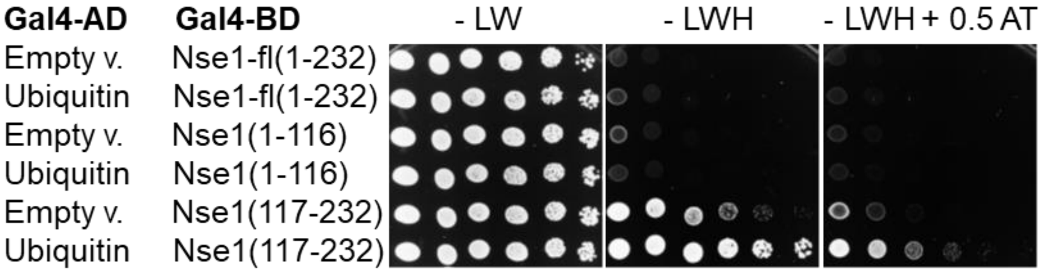

Figure S4. The C-terminal part of Nse1 interacts with ubiquitin in the yeast two-hybrid assay. Plasmids carrying indicated genes or corresponding empty vectors were co-transformed into the *S. cerevisiae* PJ69–4a strain, grown to OD600 ~ 1, fivefold serially diluted and spotted on solid media lacking leucine (L), tryptophan (W), histidine (H), in absence or presence of 0.5 mM 3-aminotriazole (0.5 AT) as depicted. Cells were grown for 3 days at 30 °C and scanned.

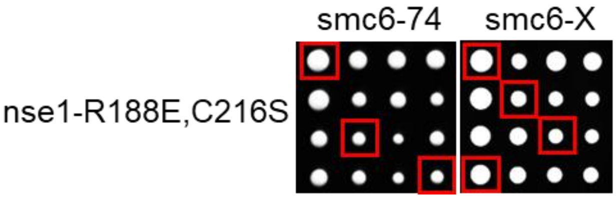

Figure S5. The synthetic lethality and growth defect observed in the *smc6-74 nse1-R188E* and *smc6-X nse1-R188E* strains, respectively, are suppressed by the *nse1-C216S* mutation. Tetrad dissection analysis of *S. pombe* diploid strains resulted from crosses between the *nse1-R188E-C216S* strain and *smc6-74* or *smc6-X*. Red rectangles mark triple mutants.

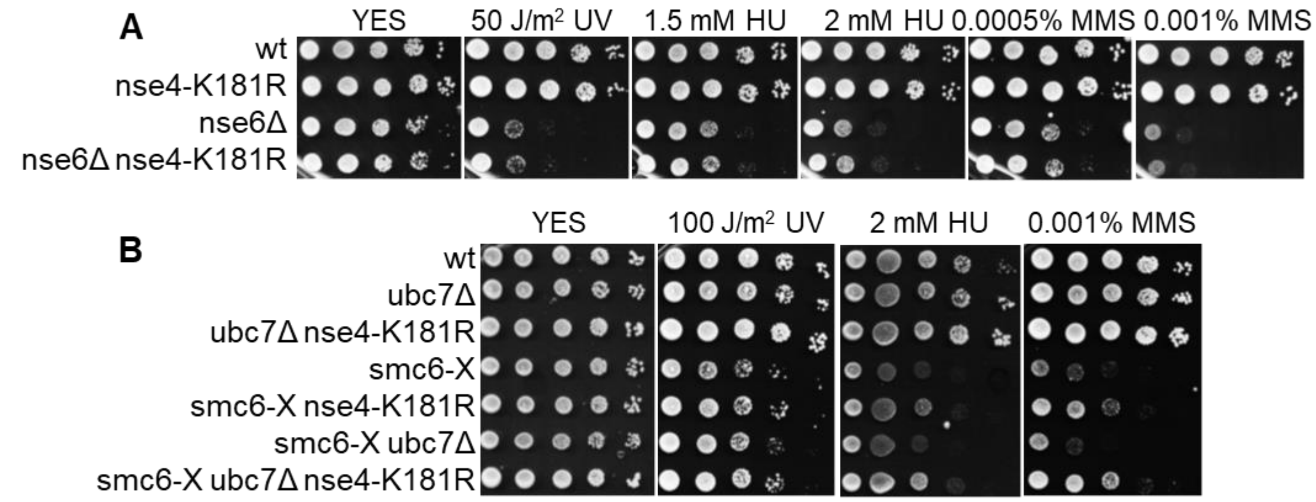

Figure S6. *Nse4-K181R* does not suppress the sensitivity of *nse6Δ*, but suppresses *smc6-X* in the absence of *Ubc7*. (**A**) *Nse4-K181R* does not suppress the sensitivity of *nse6Δ* to DNA-damaging agents. (**B**) *Nse4-K181R* suppresses the *smc6-X* sensitivity also in the absence of *Ubc7*. The depicted strains were grown to OD_{600} ~ 1, tenfold serially diluted, spotted onto rich media with the indicated amounts of HU, MMS or UV dose, and incubated at 28 °C for 3 days.

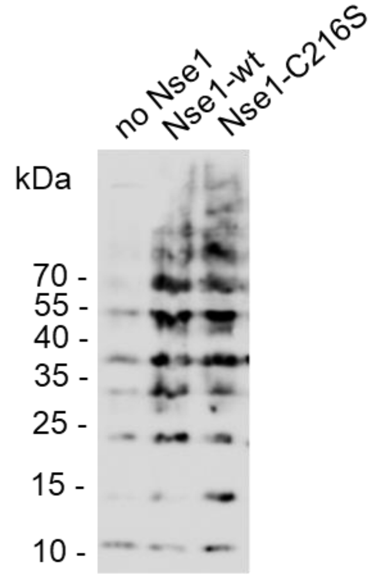

Figure S7. The C216S mutation does not inhibit Nse1 ubiquitin ligase activity. E1, Ubc13/Mms2 and biotinylated ubiquitin were incubated with ATP and $MgCl_2$ in the absence or presence of Nse1 or its C216S mutant for 1h at 37°C. The mixture was analyzed by 12% SDS–PAGE followed by western-botting and visualization of biotinylated ubiquitin using Streptavidin-HRP.
